# A new lernaeopodid genus (Copepoda, Siphonostomatoida) parasitizing *Macrourus holotrachys* (Gadiformes) from deep waters in the southeastern Pacific Ocean, based on molecular and morphological analyses

**DOI:** 10.3897/zookeys.1291.195637

**Published:** 2026-09-04

**Authors:** Raúl Castro-Romero, Martin M. Montes, Luis A. Ñacari, Fabiola Sepulveda, Marcelo E. Oliva

**Affiliations:** 1 Departamento de Ciencias Acuáticas y Ambientales, Facultad de Ciencias del Mar, Universidad de Antofagasta, Angamos 601, Antofagasta 1240000, Chile Departamento de Ciencias Acuáticas y Ambientales, Facultad de Ciencias del Mar, Universidad de Antofagast Antofagasta Chile; 2 Centro de Estudios Parasitológicos y Vectores (CEPAVE), Consejo Nacional de Investigaciones Científicas y Técnicas, Universidad Nacional de La Plata (CCT, CONICET-UNLP), Boulevard 120 s/n e/60 y 64, C.P. 1900, La Plata, Buenos Aires, Argentina Centro de Estudios Parasitológicos y Vectores (CEPAVE), Consejo Nacional de Investigaciones Científicas y Técnicas, Universidad Nacional de La Plata (CCT, CONICET-UNLP) La Plata Argentina; 3 Instituto Ciencias Naturales Alexander von Humboldt, Facultad de Ciencias del Mar y Recursos Biológicos, Universidad de Antofagasta, Angamos 601, Antofagasta 1240000, Chile Instituto Ciencias Naturales Alexander von Humboldt, Facultad de Ciencias del Mar y Recursos Biológicos, Universidad de Antofagasta Antofagasta Chile

**Keywords:** 28S rDNA, bigeye grenadier, COI mtDNA, deep-sea fishes, integrative taxonomy, lernaeopodid, new species, southeastern Pacific Ocean

## Abstract

A new genus and species of *Clavella*-like specimens parasitic on *Macrourus holotrachys*, previously reported as a host for *Clavella adunca*, clearly differ from the latter in the position and condition of the antennal sympod and rami, which are uniramous in *C. adunca* but biramous in the new species. Additional morphological traits, such as antennule armature, maxillary size, and the absence of basal swelling on the cephalothorax, support these differences. Molecular analyses corroborate these distinctions. The 28S rDNA sequences of *Neoclavella holotrachia***gen. nov. et sp. nov**. show genetic divergence of 4.1–4.7% and 6.2–6.8% from those of *C. adunca* and Lernaeopodidae gen. sp., respectively, all of which cluster within a strongly supported clade. *Clavellomimus macruri* exhibits sequence divergence with *Clavellotis girellae* (15.3%) and with *Clavellotis dilatata* (15.5%), although the corresponding nodes in the phylogram are weakly supported. COI mtDNA sequences of *Neoclavella holotrachia***gen. nov. et sp. nov**. grouped together with *Clavella* spp. in a strongly supported node, with genetic distances ranging from 17.3% to 18.2% between the new genus and the other species. These results suggest that *Macrourus holotrachys* and possibly other *Macrourus* species are not hosts for *C. adunca*, as previously reported. Alternatively, *M. holotrachys* may be parasitized by both *C. adunca* and the new species. These findings suggest that some previous records of *C. adunca* from fishes off Chile may have been misidentified.

## Introduction

Members of the family Lernaeopodidae parasitizing deep-sea fishes (>200 m) include representatives of the genera *Clavella* Oken, 1815; *Clavellomimus* Kabata, 1969; *Kabatahoia* Kazachenko, 2001; *Lernaeopoda* Wilson, 1915; *Lernaeopodina* Wilson, 1915; *Naobranchia* Hesse, 1863; *Neoalbionella* Özdikmen, 2008; *Nudiclavella* Ho, 1975; *Parabrachiella* Wilson, 1915; *Praeclavella* Castro-Romero, Montes & Martorelli, 2022; *Vanbenedenia* Malm, 1860; and *Schistobrachia* Kabata, 1964 (Ho 1975, 1985, 1993; Kabata 1979a, 1988; Boxshall 1998; Benz and Izawa 1990; Castro 1994; Castro and Gonzalez 2009; Klimpel et al. 2009; Castro-Romero et al. 2022). These genera belong to most of the clades delimited by Kabata (1979a). One of these clades is the *Clavella* branch, which currently comprises 20 genera, including recently revised taxa and new combinations (*Praeclavella* and *Maxiclavella*) as well as *Simplalella*, which was separated from *Alella* (Castro-Romero et al. 2022, 2025a).

The study of parasites from the bigeye grenadier *Macrourus holotrachys* Günther, 1878, a deep-water fish from the southeastern Pacific Ocean, allowed for the collection of *Clavella*-like specimens. These specimens were analyzed morphologically and genetically (28S rDNA and COI mtDNA) to clarify their phylogenetic relationships.

Previously recorded copepods from grenadiers (*Macrourus* spp.) include *Clavella adunca* (Type locality. Tocopilla, northern Chile (22°30'S, 70°40'W), depth 1,000–2,200 m., 1762), *Clavella zini* Kabata, 1979, and *Clavellomimus macruri* (Hansen, 1923). *Clavella adunca* has been reported from deep-sea fishes (Boxshall 1998; Klimpel et al. 2009; Münster et al. 2016), parasitizing *Macrourus berglax* Lacepède, 1801, *M. holotrachys*, and *M. whitsoni* (Regan, 1913). *Clavella adunca* has been described as a parasite of members of many families occurring in both deep-water and coastal habitats. Most records are from the North Atlantic and North Pacific, with additional reports from Japan and Korea, as well as from the South Pacific and Antarctic waters (Table 1). *Clavella adunca* also has a long list of synonyms (Walter and Boxshall 2025), and its true occurrence across different hosts and localities requires verification. *Clavellomimus* comprises four species, but only *Clavellomimus macruri* has been reported from deep-water fish, specifically *M. berglax* (Klimpel et al. 2009).

**Table 1.** Recorded hosts species for *Clavella adunca*.

**Host**	**Locality**	**Reference**
Anarhichadidae		
*Anarhicas lupus* L.	Scotian Shelf (N Atlantic)	Hogans (1995)
Callionymidae		
*Callionymus lyra* (Bloch)	Great Britain (N Atlantic)	Ho (1977)
Clupeidae		
Gadidae		
*Boreogadus saida* Lepechin	Spitsbergen and Barent Sea, Northeastern Greenland, Canada, Russian Artic, Alaska (Arctic Ocean)	Schram (1980); Gjøsæter (1987); Køie (2009); Heegaard (1945)
*Gadus macrocephalus* Tilesius	Alaska (N Pacific)	Wilson (1920); Kabata (1979a, 1988); Moles (1982); Ho (1977)
*Gadus morhua* (L.) Syn: *Gadus callarias*	British Isle, Iceland, David strait, Nova Scotia shelf and southern Gulf of St. Lawrence (N Atlantic)	Janusz (1979); Kabata (1979a, 1988); Ho (1977); Appy and Burst (1982); Redkozubova (1976); Morrison (1988)
*Gazza minuta* (Bloch)	Off Sri Lanka (Indian Ocean)	Kabata (1979a)
*Hexagrammus octogrammus* (Pallas)	Japan (N Pacific)	Kabata (1979b); Ho (1977)
*Melanogrammus aeglefinus* (L.)	Scotian Shelf (N Atlantic), NW Atlantic	Kabata (1979a, 1988); Scott (1981);
*Merlangius merlangus* (L.)	North Sea	Potter et al. (1988); Kabata (1979a); Ho (1977)
*Microgadus proximus* (Girard)	British Columbia	Kabata (1979b, 1988); Ho (1977)
*Pollachius pollachius* (L.)	North Sea	Kabata (1979a); Ho (1977)
*Pollachius virens* (L.)	Off Iceland, Skagerrak, NW Atlantic	Kabata (1979a, 1988); Ho (1977);
*Trisopterus luscus* (L.)	North Sea	Ho (1977); Kabata (1979a)
*Theragra chalcogramma* (Pallas)	Alaska	Ho (1977)
Kyphosidae		
*Girella fasciata* (Kner & Steindachner)	West coast of South America	Wilson (1923)
Hexagrammidae		
*Pleurogrammus* sp.	Sea of Japan	Ho (1977)
Leiognastidae		
*Gaza minuta* (Bloch)	Greenland	Ho (1977)
Macrouridae		
*Coryphaenoides rupestris* Gunnerus	NW Atlantic	Kabata (1979b); Zubchenko (1981)
*Coryphaenoides* sp.	South Pacific, Chile	Castro (1994)
*Macrourus berglax* (Syn: *Coryphaenoides berglax* (Lacepede); *Macrourus fabricii*)	Davis Strait, Irminger Sea, east Greenland Sea, Flemish Cap. S. Labrador.	Ho (1977); Ho (1985); Palm and Klimpel (2008); Zubchenko (1981); Klimpel et al. (2006)
*Macrourus carinatus* (Gunther)	Falkland Islands (South Atlantic)	Gaevskaya and Rodjuk (1988)
*Macrorurus holotrachys* (Gunther) (Syn: *Coryphaenoides holotrachys*)	Heart Island Shelf (Antarctica)	Rohde et al. (1998)
*Macrourus whitsoni* (Regan)	Off Antarctic; Halley Bay, Shetland Islands (Antarctica)	Ho (1977); Walter et al. (2002); Kabata and Gusev (1966); Kabata (1979a)
Nothothaenidae		
*Eleginops maclovinus* (Cuvier)	Canal Tenglo, Bahía Codihue and San Ignacio, Chile (S Pacific)	Henriquez et al. (2011)
*Trematomus loenbergi* Regan	East Antarctica	Ho (1977); Kabata (1979a)
*Patagonotothen ramsayi* (Regan)	Patagonian waters (S Atlantic)	Cantatore et al. (2012)
Pholidae		
*Pholis gunnellus* (L.)	Mediterranean	Ho (1977)
Psychrolutidae		
*Malacocottus zonurus* Bean	Eastern Bering Sea, Kuril Islands	Ho et al. (2005)
Sebastidae		
*Sebastes melanops* Girard	Korea	Chun (2002, 2003)
*Sebastes marinus*	Davis Strait (N Atlantic)	Ho (1977)
Sparidae		
*Diplods sargus sargus* (L.) *Syn*: *Sargus rondeleti*	Mediterranean	Ho (1977)
Merluccidae		
*Merluccius merluccius* (L.)	Adriatic Sea	Ho (1977)
Somniosidae		
*Somniosus micerocephalus* (Bloch & Schneider)	Off Iceland, Greenland	Ho (1977)
Zoarcidae		
*Licenchelys paxillus* (Gode & Beare)	Atlantic	Kabata (1979b, 1988)
*Licodes lavalei* Vladykov & Tremblay	Massachusetts (N Atlantic)	Ho (1977)

The 28S rDNA gene has proven useful for elucidating phylogenetic relationships among related species (Olsen and Woese 1993; Gonzalez et al. 2016). COI mtDNA is a suitable marker for species delimitation, helps clarify species boundaries, and has been widely used for parasitic copepods (Dippenaar et al. 2010; Muñoz et al. 2015; Castro-Romero et al. 2016, 2022; González et al. 2016; Montes et al. 2017).

The aim of this study was to describe, based on morphology and molecular analyses, a new genus and species of copepod parasitizing *Macrourus holotrachys* in the southeastern Pacific Ocean and its relationships with other members of the family Lernaeopodidae.

## Materials and methods

### Collection of samples and morphological study

During 2015, a total of 30 specimens of the bigeye grenadier *Macrourus holotrachys* were obtained as by-catch from the Patagonian toothfish *Dissostichus eleginoides* fishery in northern and central Chile. Fishes were frozen on board (–18 °C) and transported to the parasitological laboratory at the University of Antofagasta. After thawing, the fish were examined for parasitic copepods. Recovered copepods were fixed in 96% ethanol for morphological and molecular analyses. The specimens were examined under a stereomicroscope (Olympus, Japan), and the appendages were dissected after clearing in lactic acid when necessary. Drawings were made with a camera lucida. Measurements (in micrometers) were taken using a reticulated eyepiece and are presented as the mean and range. Terminology follows Kabata (1979a) for lernaeopodid morphology and Huys and Boxshall (1991) for the maxillule. The prevalence (%) of infected fish was estimated, as well as the mean intensity of infection (mean number of parasites per infected host) (Bush et al. 1997).

### Molecular analysis

A tissue sample of 2–3 mm^³^ preserved in ethanol was used for DNA extraction, together with 5 μL of insect lysis buffer and 0.5 μL of proteinase K (20 mg/mL), in each well of a 96-well Eppendorf plate. Genomic DNA was extracted using the glass-fiber (GF) protocol for 96-well plates (Ivanova et al. 2006a, 2006b).

Amplification of the 5' barcode region of COI was performed using the primers HCO2198_t1 (5’-CAG GAA ACA GCT ATG ACT AAA CTT CAG GGT GAC CAA AAA ATC A-3’) and LCO1490_t1 (5’-TGT AAA ACG ACG GCC AGT GGT CAA ATC ATA AAG ATA TTG G-3’) (Folmer et al. 1994). The 28S rDNA region was amplified using the primers 28S-F (5'-ACA ACT GTG ATG CCC TTA G-3') and 28S-R (5'-TGG TCC GTG TTT CAA GAC G-3') (Song et al. 2008).

PCRs were carried out in 96-well plates. The reaction mixture contained 625 μL of 10% trehalose, 200 μL of water, 125 μL of buffer, 62.5 μL of MgCl_2_ (50 mM), 6.25 μL of dNTPs (10 mM), 12.5 μL of each primer (10 µM), and 6 μL of Taq DNA polymerase (5 U/mL). Each well contained 10.5 μL of mixture and 2 μL of genomic DNA. The PCR profile included an initial step at 95 °C for 2 min, followed by 5 cycles of 94 °C for 30 s, annealing at 45 °C for 40 s, and extension at 72 °C for 1 min; then, 35 cycles of 94 °C for 30 s, annealing at 51 °C for 40 s, and extension at 72 °C for 1 min, with a final extension at 72 °C for 10 min. Amplicons were visualized on a 2% agarose E-Gel™ 96-well system (Invitrogen).

Sequencing was performed by Macrogen (Korea). Sequencing reactions were carried out in an MJ Research PTC-225 Peltier thermal cycler using ABI PRISM BigDye™ Terminator Cycle Sequencing Kits with AmpliTaq DNA polymerase (FS enzyme) (Applied Biosystems) following the manufacturer’s instructions. Single-pass sequencing was performed on each template using universal primers. Fluorescently labelled fragments were purified from unincorporated terminators using the BigDye XTerminator purification protocol. The samples were subsequently resuspended in distilled water and subjected to electrophoresis in an ABI 3730xl sequencer (Applied Biosystems). The COI barcode sequences were obtained following Ivanova and Grainger (2007).

The sequences were assembled using Geneious Pro v. 10 (http://www.geneious.com; Kearse et al. 2012). BLASTn searches were conducted in GenBank to identify homologous sequences for comparison (Table 2). The sequences of each gene were aligned separately using MAFFT v. 7 (Katoh and Standley 2013). The COI mtDNA alignment was checked for pseudogenes in Geneious by translating sequences using the invertebrate mitochondrial genetic code. The best partitioning scheme and substitution model for 28S rDNA and COI mtDNA were selected under the Bayesian information criterion (BIC; Schwarz 1978) using the greedy search strategy in PartitionFinder v. 2.1.1 (Lanfear et al. 2017).

**Table 2.** Collection data and GenBank accession numbers of the copepods analyzed in this study. Newly generated sequences are shown in bold.

**Copepod parasite species**	**GenBank accession**		**Host (Family)**	**Locality**	**Reference**
	**28S rDNA**	**COI**			
Lernaeopodidae					
*Alella canthari* (Heller, 1865)	-	PX243275, PX243276	*Spondyolosoma cantharus* Linnaeus (Sparidae)	Turkey	Castro-Romero et al. (2025a)
*Alella macrotrachelus* (Brian, 1906)		PX243277, PX243278	*Diplodus vulgaris* (Forster) (Sparidae)	Turkey	Castro-Romero et al. (2025a)
*Clavella adunca* (Strøm, 1762)	MF077820	-	Unknown	North Sea	Khodami et al. (2017)
*Clavella adunca* (Strøm, 1762)	-	KT208702, KT208865, KT208899	*Gadus morhua* Linnaeus (Gadidae)	North Sea	Raupach et al. (2015)
*Clavella stellata* (Krøyer, 1838)	DQ180339	-	Unknown	Unknown	Tjensvoll et al. unpublished
*Clavella perfida* Wilson C.B., 1915	-	LC179744–LC179746	*Gadus chalcogrammus* Pallas	Japan	Yanagimoto and Ichimura (2018)
*Clavellisa ilishae* Pillai, 1962	-	ON023682	Unknown	India	Nikhila and Sudha unpublished
*Clavellisa obcordatus* Rangnekar, 1957	-	ON023683	Unknown	India	Nikhila and Sudha unpublished
*Clavellisa scombri* (Kurz, 1877)	-	PV696745, PV696746	*Scomber scombrus* Linnaeus (Scombridae)	Turkey	Castro-Romero et al. (2025b)
***Clavellomimus macruri* (Hansen, 1923)**	**MH917255**	-	***Macrourus holotrachys* Günther (Macrouridae)**	**Chile**	**This study**
*Clavellotis dilatata* (Krøyer, 1963)	KX815915	KX815897–KX815900	*Cheilodactylus variegatus* Valenciennes (Cheilodactylidae)	Chile	Castro-Romero et al. (2022)
*Clavellotis girellae* Castro et al., 2025	PV698387	PV696726	*Girella laevifrons* (Tschudi) (Kyphosidae)	Chile	Castro-Romero et al. (2025b)
*Clavellotis dubius* Castro et al., 2025	-	PV696741	*Chromis crusma* (Valenciennes) (Pomacentridae)	Chile	Castro-Romero et al. (2025b)
*Clavellotis fallax*	-	PV696731	*Dentex dentex* (Linnaeus) (Sparidae)	Turkey	Castro-Romero et al. (2025b)
*Clavellotis sebastidis* Castro & González, 2005	-	PV696735	*Sebastes oculatus* (Valenciennes) (Sebastidae)	Chile	Castro-Romero et al. (2025b)
*Clavellotis strumosa* (Brian, 1906)	-	PV696734	*Pagrus pagrus* (Linnaeus) (Sparidae)	Turkey	Castro-Romero et al. (2025b)
Lernaeopodidae gen. sp.	KR048860	-	Unknown	unknown	Baek and Hwang unpublished
*Maxiclavella simplex* (Castro Romero & Baeza Kuroki, 1985)	KX815911	KX815901–KX815993	*Isacia conceptionis* Cuvier (Haemulidae)	Chile	Castro-Romero et al. (2022)
***Neoclavella holotrachia* gen. nov. et sp. nov**.	**MH917260 MH923194**	**MH932105**	***Macrourus holotrachys* Günther (Macrouridae)**	**Chile**	**This study**
*Parabrachiella anisotremi* (Castro-Romero & Baeza-Kuroki, 1989)	KX815913		*Anisotremus scapularis* Tschudi (Pomadasidae)	Chile	Castro-Romero et al. (2022)
*Parabrachiella auriculata* Castro-Romero & Baeza Kuroki, 1987	KX815914		*Cilus gilberti* Abbott (Sciaenidae)	Chile	Castro-Romero et al. (2022)
*Parabrachiella hugu* (Yamaguti, 1939)	KR048861		Unknown	South Korea	Baek and Hwang unpublished
*Parabrachiella merluccii* (Bassett-Smith, 1896)	DQ180347	-	Unknown	Unknown	Tjensvoll et al. unpublished
*Praeclavella applicata* (Castro Romero & Baeza Kuroki, 1985)	KX815909	KX815891	*Anisotremus scapularis* Tschudi (Pomadasidae)	Chile	Castro-Romero et al. (2022)
*Praeclavella caudata* (Castro-Romero & Baeza-Kuroki, 1985)	KX815910	KX815895	*Anisotremus scapularis* Tschudi (Pomadasidae)	Chile	Castro-Romero et al. (2022)
*Praeclavella nasalis* Castro-Romero, Montes & Martorelli, 2022	KX815912	KX815904	*Isacia conceptionis* Cuvier (Haemulidae)	Chile	Castro-Romero et al. (2022)
*Simplalella igillimpethu* (*=Alella igillimpethu* Erasmus, Hadfield, Wepener & Smit, 2022)	OP548636	OP548139	*Clinus superciliosus* (Linnaeus) (Clinidae)	South África	Erasmus et al. (2023); Castro-Romero et al. (2025a)
Caligidae					
*Caligus cheilodactyli* Kroyer, 1863*	KU317548	KU317605	*Sebastes oculatus* Valenciennes (Sebastidae)	Chile	González et al. (2016)

*Outgroup.

The selected substitution model for 28S rDNA was HKY+G, and for COI, the dataset was partitioned by codon position: GTR+I+G (first), GTR+I+G (second), and GTR+I+G (third). *Caligus cheilodactyli* Krøyer, 1863, was used as the outgroup.

Phylogenetic reconstruction was performed using Bayesian inference (BI) in MrBayes v. 3.2.3 (Ronquist et al. 2012). Two parallel analyses of the Metropolis-coupled Markov chain Monte Carlo (MCMC) method were run for 20 million generations, with sampling every 1,000 generations. The average standard deviation of split frequencies was less than 0.01 at the end of the run. Clade robustness was assessed by Bayesian posterior probabilities (PPs), with PPs > 95% considered strongly supported. Majority-rule consensus trees with branch lengths were reconstructed after discarding the first 25% of the sampled trees as burn-in.

Uncorrected *p* distances were calculated using pairwise deletion in MEGA X (Kumar et al. 2018).

Type and voucher specimens were deposited in the Museo Nacional de Historia Natural, Santiago, Chile.

## Results

### Taxonomy

**Family Lernaeopodidae Milne Edwards, 1840**

#### Neoclavella gen. nov.

Taxon classificationAnimaliaGadiformesLernaeopodidae

3799D751-6343-51D9-AFE7-43F5E4C57DCF

https://zoobank.org/B72B9312-32BE-463B-B6F8-EE08280CA61A

##### Type species.

*Neoclavella holotrachia* sp. nov.

##### Generic diagnosis.

Female. *Clavella*-like. The cephalothorax is straight and not deflected on the trunk; the base is not swollen. The antennule is apparently 2-segmented, with a broad base; no whip or solus is present. The distal armature has five elements: a short gibber bearing bifid seta 5; seta 3 with a broad base; and short setae 4, 2, and 1. Seta 6 is absent. The antenna is biramous; the endopod and exopod are well developed and perpendicular to the sympod. The mandible lacks secondary dentition; the dentition formula is P1, P1, P1, P1, P1 (the last two are longer), B2. The maxillule has an endite bearing two papillae, each with one seta; the palp is laterally located and armed with two short, equal setae. The maxillae are fused. The bulla is cup-shaped. The maxilliped has a robust corpus bearing a subcircular protrusion armed with a row of spinules and a short spine. The claw is short, with an annexed barb and two basal spinules; additional spinules are present on the distal shaft. A genital process is present.

##### Etymology.

The generic name *Neoclavella* refers to the novelty of the genus within the *Clavella* branch and the biramous antenna, in contrast to the uniramous condition in related species formerly included in *Clavella*. Gender feminine.

#### Neoclavella holotrachia sp. nov.

Taxon classificationAnimaliaGadiformesLernaeopodidae

9A555A55-17A0-5E3F-9DDB-1DAE5DE1B5E2

https://zoobank.org/568BF9A9-7FC4-4F1A-88AE-60AF6C28D069

Figs 1, 2, 3

##### Type host.

*Macrourus holotrachys* Günther, 1878 (Macrouridae).

##### Site of infection.

Gills.

##### Type locality.

Tocopilla, northern Chile (22°30'S, 70°40'W), depth 1,000–2,200 m.

##### Other localities.

Iquique, northern Chile (20°15'S, 70°40'W); Constitución, central Chile (35°15'S, 73°05'W), Chile.

##### Prevalence.

53.3%. (16 infected specimens from a sample of 30 specimens).

##### Mean intensity.

3.7.

##### Type material.

***Holotype*** ♀. MNHNCL COP-15172 (Museo Nacional de Historia Natural, Chile). ***Paratypes*** 3 ♀♀ MNHNCL COP 15173-15175.

##### Representative DNA sequences.

GenBank accession numbers:

28S rDNA: MH917260, MH923194.

COI mtDNA: MH932105.

##### Description.

**Figure 1.**
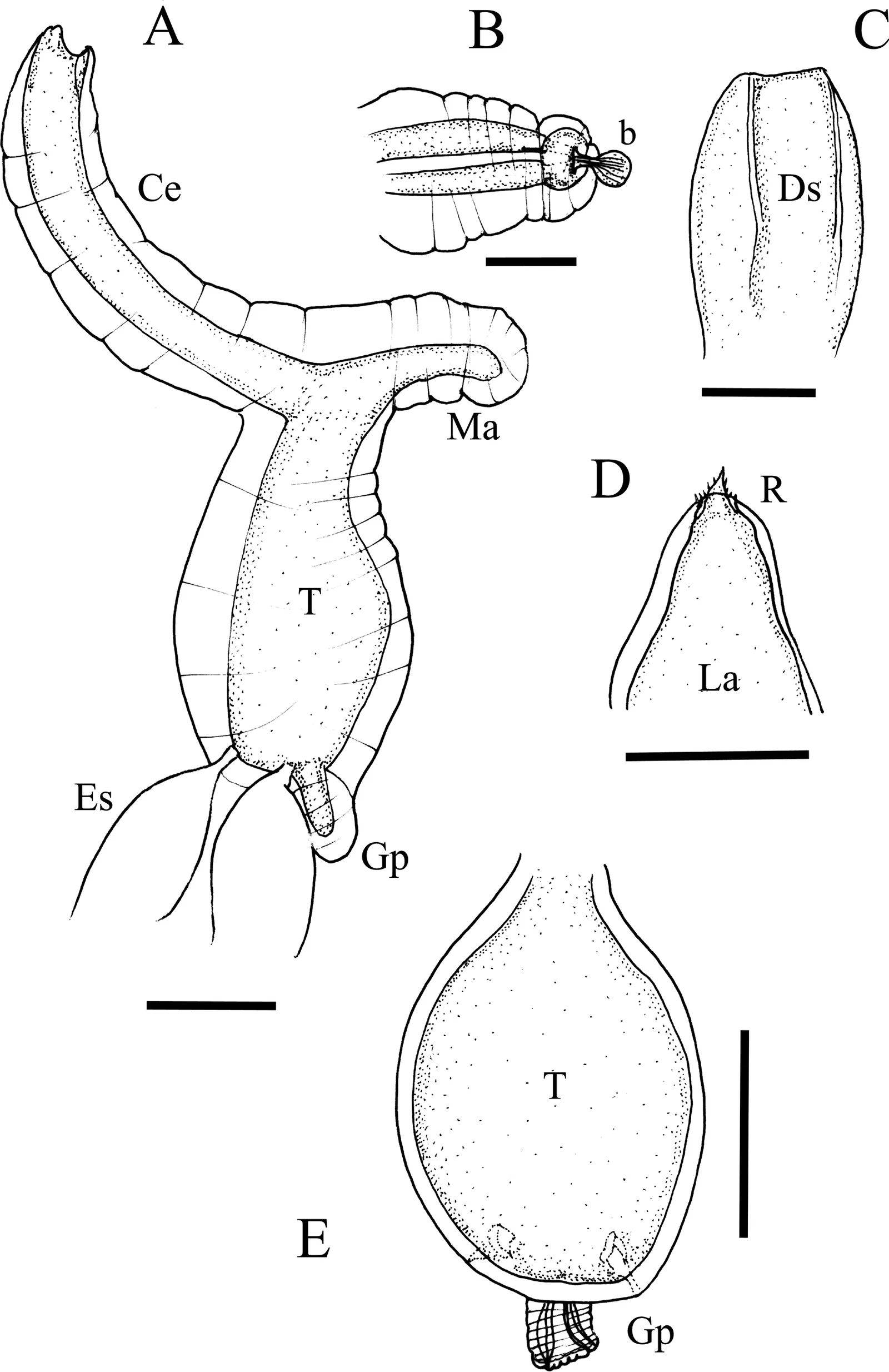
*Neoclavella holotrachia* gen. nov. et sp. nov. **A**. Female lateral view; **B**. Maxilla ventral view; **C**. Dorsal shield, dorsal view; **D**. Labrum and rostrum; **E**. Trunk, dorsal view. Abbreviations: b = bulla, Ce = cephalothorax, Ds = dorsal shield, Es = egg sac, Gp = genital process, Ma = maxilla, La = labrum, R = rostrum, T = trunk. Scale bars: 1500 µm (**A**), 500 µm (**B, C**), 50 µm (**D**), 1000 µm (**E**).

**Figure 2.**
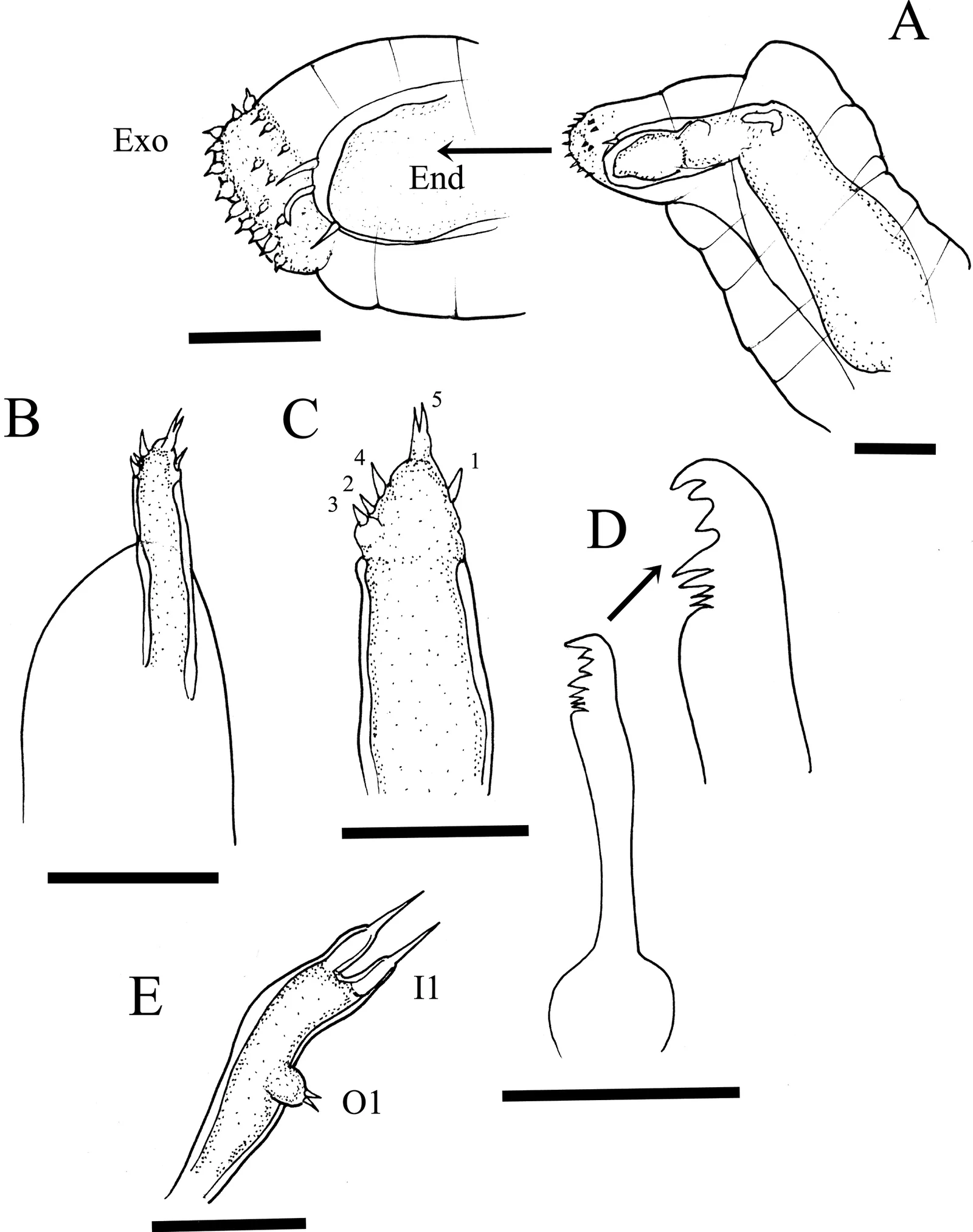
Buccal appendages of *Neoclavella holotrachia* gen. nov. et sp. nov. **A**. Antenna lateral view, and detail of the exopod and endopod; **B**. Antennule; **C**. Antennule distal segment armature; **D**. Mandible and detail of dentition; **E**. Maxillule lateral view. Abbreviations: 1, 2, 3, 4, 5 = setae names, Il = inner lobe, Ol = outer lobe, Exo = exopod, End = endopod. Scale bars: 50 µm (**A**), 25 µm (**B**), 50 µm (**C, E**), 30 µm (**D**).

**Figure 3.**
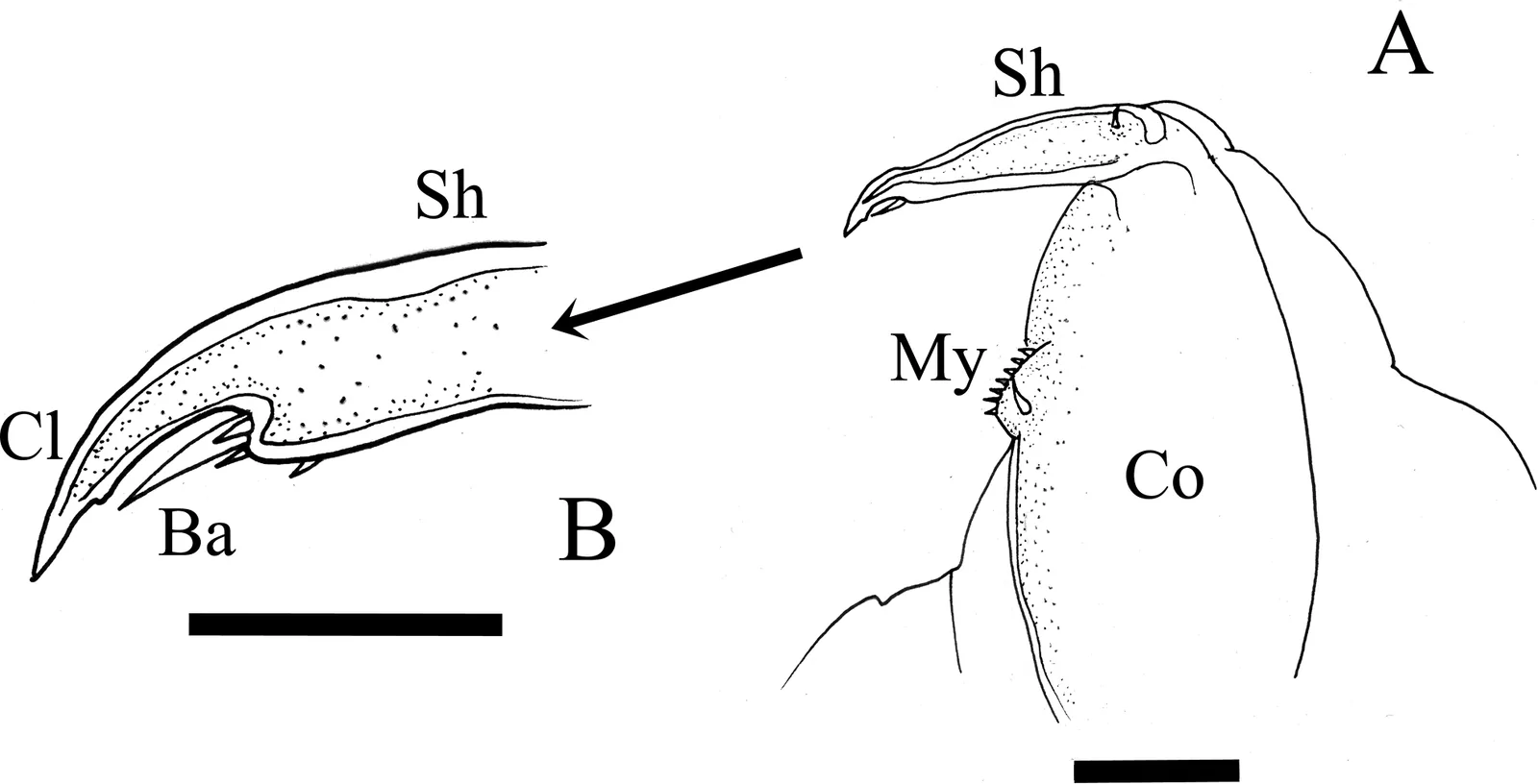
**A**. *Neoclavella holotrachia* gen. nov. et sp. nov. Maxilliped lateral view; **B**. Detail of claw armature. Abbreviations: Ba = barb, Cl = claw, Co = corpus, My = myxal area, Sh = shaft. Scale bars: 30 µm (**A**), 50 µm (**B**).

**Adult female**. Based on the holotype and seven adult females (measurements are provided in Table 3). The cephalothorax cylindrical, slightly longer than trunk (Fig. 1A). No swelling at the cephalothorax base was observed. The maxilla (Fig. 1B) of medium size, with short anchor and short, subcircular manubria. The dorsal shield with parallel sides (Fig. 1C). Labrum (Fig. 1D) is subtriangular, with a short rostrum bearing fine setules. The excretory duct is nipple-like and short. The trunk is pyriform and narrow anteriorly (Fig. 1E), bearing a subrectangular genital process. Caudal rami are absent. The Antenna (Fig. 2A) is biramous, with rami perpendicular to the sympod; the sympod is twice the length of the rami, and the exopod is longer than the endopod. The endopod (Fig. 2A, detail) is armed with three subequal setae. The antennule (Fig. 2B, C) has indistinct segmentation and is apparently 2-segmented, with distal armatures of 1, 5, 4, 2, and 3 elements, which are well separated; seta 5 is bifid on a gibber. The mandible (Fig. 2D) lacks secondary dentition and has five primary and two basal teeth. The maxillule (Fig. 2E) is bilobed and slender; the endite has two equal setae, and the palp is a short ventral papilla with two short, equal setae. The maxilliped (Fig. 3A) has a robust corpus bearing rows of spinules on a convex myxal outgrowth plus one spine. The shaft has a proximal spine. The claw (Fig. 3B) has a short tooth-like ventrodistal projection and a long-annexed barb with two basal spinules and additional spinules posterior to the base.

**Table 3.** Morphometric measurements of *Neoclavella holotrachia* gen. nov. et sp. nov. and *Clavellomimus macruri* from *Macrourus holotrachys*. L: length; W: width; n: specimens.

		***Neoclavella holotrachia* gen. nov. et sp. nov**.	***Clavellomimus macruri***
***n***		**8**	**1**
Cephalothorax	L	2.65 (2.10–3.59)	10.31
	W	0.72 (0.64–0.80)	1–00
Maxilla	L	1.01 (0.51–1.80)	1.67
	W	0.65 (0.49–0.92)	1.03
Trunk	L	2.30 (1.90–3.08)	6.15
	W	1.55 (1.33–1.95)	1.90
Genital process	L	0.58 (0.49–0.74)	
	W	0.39 (0.56–0.46)	
Egg sac	L	2.37 (1.80–3.41)	6.15
	D	0.69 (0.56–0.90)	1.03

**Male**. Unknown.

##### Etymology.

The specific name *holotrachia* refers to the host species.

##### Taxonomic remarks.

*Neoclavella* gen. nov. is similar to the genera *Clavellomimus* and *Clavella*, both of which are representatives of the *Clavella* branch of parasites found on *Macrourus holotrachys*.

*Neoclavella* gen. nov. differs from *Clavellomimus* in several morphological characteristics. The trunk in *Clavellomimus* is elongate and narrow and lacks a genital process, whereas in the new genus the trunk is suborbicular, is slightly longer than wide, and has a distinct genital process. The antennule of *Clavellomimus* has four long elements, whereas in the new genus, the elements are shorter and element 5 is situated on a gibber.

The antenna of *Clavellomimus* consists of a globose exopod ornamented with several rows of spinules and a very short endopod, oriented almost perpendicular to the exopod and aligned with the base. In contrast, in *Neoclavella* gen. nov. the exopod is perpendicular to the base, and both rami are aligned in the same plane and run parallel to each other. In both genera, the maxillule endite bears two setae; however, the palp differs, bearing a single seta in *Clavellomimus* and two setae in the new genus. Finally, *Clavellomimus* possesses separate, short maxillae with a small bulla, whereas the new genus is characterized by fused, medium-sized maxillae.

*Neoclavella* gen. nov. differs from *Clavella* primarily in possessing a biramous antenna with a well-developed exopod bearing three apical rows of spinules, whereas members of *Clavella* have a uniramous antenna with a reduced or absent exopod. Nevertheless, a comparison of *Neoclavella holotrachia* gen. nov. et sp. nov. with *Clavella adunca* is particularly relevant because of their overall morphological similarity and their shared host, *Macrourus holotrachys*.

In the new species, the cephalothorax is longer than the trunk, whereas in *C. adunca*, both regions are approximately equal in length. The dorsal shields are narrower in *N. holotrachia* gen. nov. et sp. nov. and wider in *C. adunca*. The trunk length reaches a maximum of 3,590 μm in *N. holotrachia* gen. nov. et sp. nov. compared with 7,000 μm in *C. adunca*. The trunk of the new species is oval to oblong and anteriorly narrowed, whereas it is subquadrate in *C. adunca*. The maxillae are of medium size in *N. holotrachia* gen. nov. et sp. nov. but very short in *C. adunca*.

Further differences are evident in the appendages. The antennule of the new species lacks a terminal whip, which is present in *C. adunca*; the antennular armature is shorter and more widely spaced in the species now described but compact in *C. adunca*. The antenna is biramous in *N. holotrachia* gen. nov. et sp. nov. but uniramous in *C. adunca*; in the new species, the exopod is oriented perpendicular to the sympod, while in *C. adunca*, it is aligned with it. In the new species, the antennal endopod bears three elements, compared with four in *C. adunca*.

The mandible of the species now described lacks secondary teeth, which are present in *C. adunca*. The endite and palp lobes of the maxillule, bear two setae and lack spinules in *N. holotrachia* gen. nov. et sp. nov. whereas *C. adunca* possesses three setae and distinct denticles. The myxal area of the maxilliped in *N. holotrachia* gen. nov. et sp. nov. exhibits a prominent convex projection armed with a row of denticles, in contrast to the smaller denticulate pad present in *C. adunca*. The claw base has three spinules in *N. holotrachia* gen. nov. et sp. nov. but several rows of spinules are present in *C. adunca*.

In addition, the recently described genus *Praeclavella*Castro-Romero et al. (2022), which belongs to the *Clavella* branch, also possesses biramous antennae; however, in that genus, both rami are aligned with the sympod, whereas in *Neoclavella* gen. nov. they are perpendicular.

###### *Clavellomimus* Kabata, 1969

#### Clavellomimus macruri

Taxon classificationAnimaliaSiphonostomatoidaLernaeopodidae

(Hansen, 1923)

92EB7699-B062-57D1-9667-4EBC8781348F

Figs 4, 5

Clavella macruri Hansen, 1923: PI. IV, figs 6a–f.

##### Type host.

*Macrourus holotrachys* Günther, 1878 (Macrouridae).

##### Site of infection.

Fins.

##### Localities.

Tocopilla, northern Chile (22°30'S, 70°40'W), Constitución, central Chile (35°15'S, 73°05'W), Chile.

##### Prevalence.

10.0%.

##### Mean intensity.

1.0.

##### Material.

Voucher specimen: 1♀ MNHNCL COP 15176 (Museo Nacional de Historia Natural, Chile).

##### Representative DNA sequences.

GenBank accession number 28S rDNA: MH917255.

##### Description.

**Figure 4.**
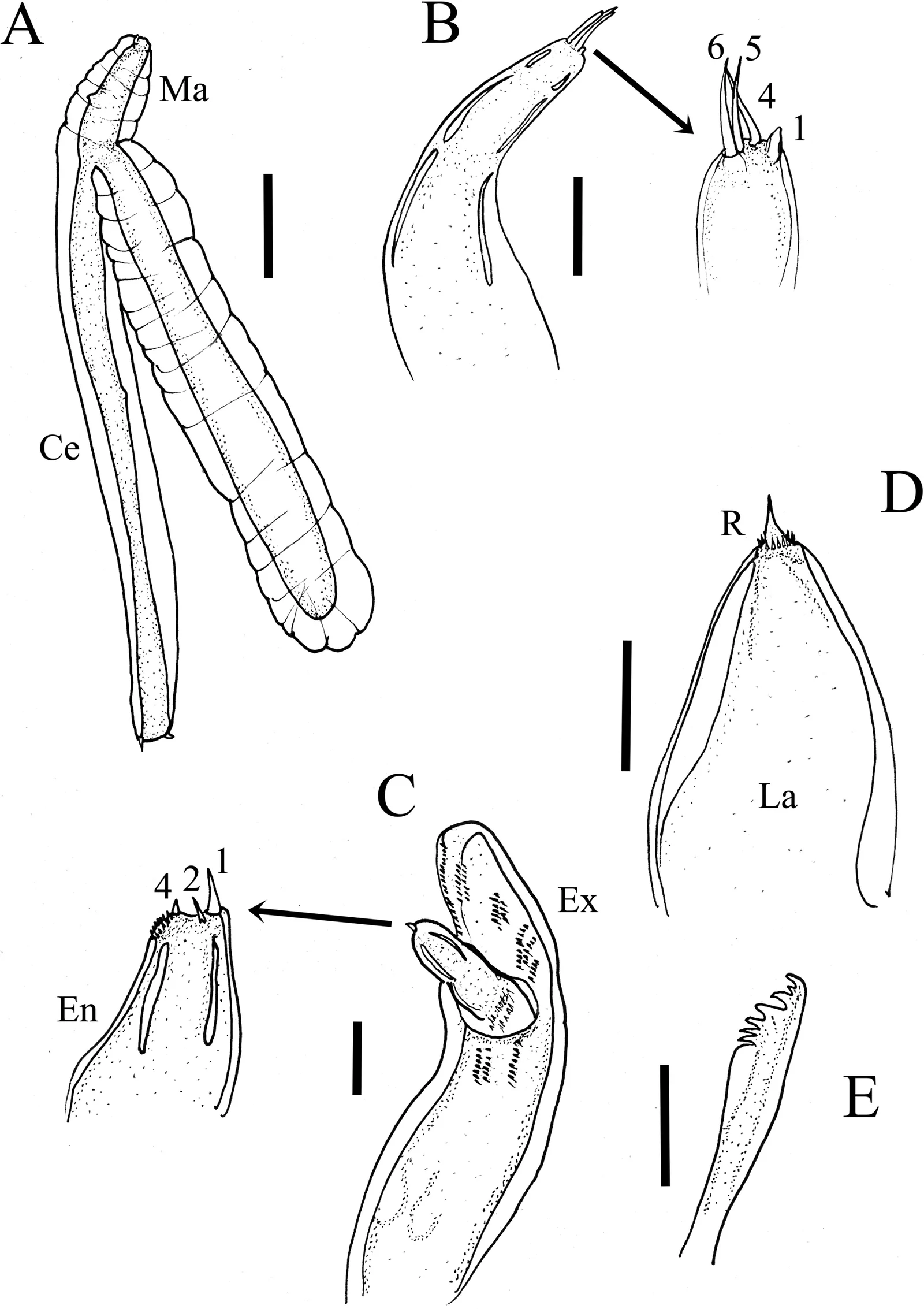
*Clavellomimus macruri*. **A**. Female lateral view; **B**. Antennules and details of distal armature; **C**. Antenna and detail of endopod armature; **D**. Labrum and rostrum; **E**. Mandible. Abbreviations: 1, 2, 4, 5, 6 setae names, En = endopod, Ex = exopod, Ma = maxilla, La = labrum, R = rostrum. Scale bars: 100 µm (**A**), 50 µm (**B–E**).

**Figure 5.**
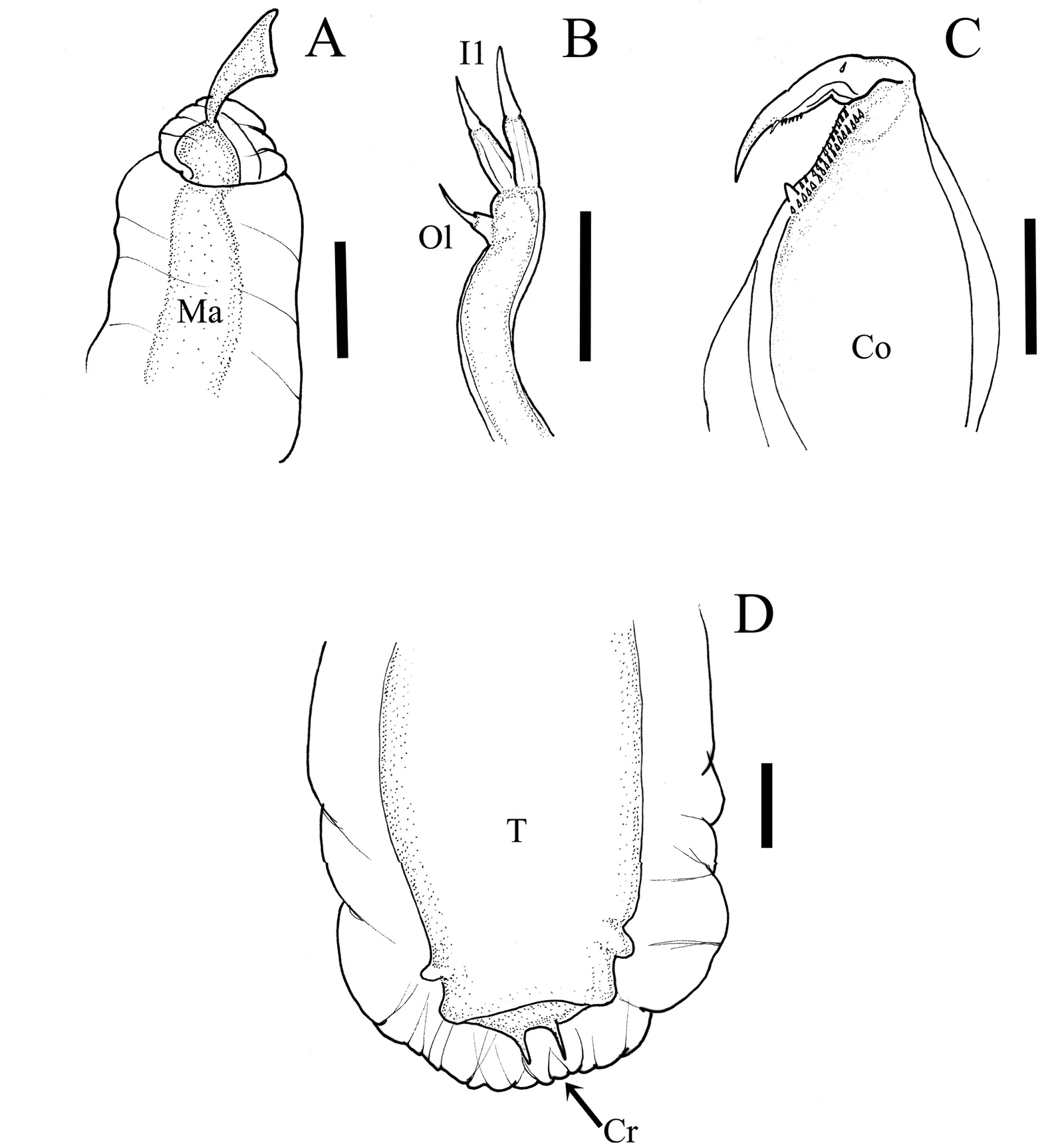
*Clavellomimus macruri*. **A**. Bulla; **B**. Maxillule; **C**. Maxilliped; **D**. Trunk distal part. Abbreviations: Co = corpus, Cr = caudal rami, Ma = maxilla, Il = inner lobe, Ol = outer lobe, T = trunk. Scale bars: 50 µm (**A**), 100 um (**B, C**), 500 µm (**D**).

**Adult female**. Measurements are given in Table 3. Based on one ovigerous female. The cephalothorax (Fig. 4A) longer than the trunk, with the latter being approximately 60% of the cephalothorax length. Dorsal shield present and the maxilla is medium-sized and broad (16% of the cephalothorax length), the arms are slightly separated and covered by a cuticle. The trunk is cylindrical and narrow (Fig. 4A), with a short lateral projection and a posterior lobule for the genital opening and lacks a genital process. The antennule (Fig. 4B) is apparently 3-segmented; the basal segment is the largest, and the second is longer than the third. The distal armature has three subequal setae (22 μm) and a short lateral spine; no solus or whip was detected. In the antenna (Fig. 4C, detail), the sympod and exopod are aligned; the exopod is elongate, and the distal surface has pads of small spinules, with additional pads located more basally. The endopod is perpendicular to the exopod and has one spiniform process (1), an inner seta (3), a short outer apical process, and a pad of spinules distally. The labrum (Fig. 4D) is long, with a short distal rostrum and rows of basal setules. The mandible (Fig. 4E) has a secondary tooth; the formula is P1, S1, P1, P1, and B4. The maxilla (Fig. 5A) has fused arms of medium size; each arm is distally suborbicular and lateral to the bulla origin. The bulla has a slender manubrium and a suboblique anchor. The maxillule (Fig. 5B) is bilobed; the endite has two long setae, and the palp has one long ventral seta. The maxilliped (Fig. 5C) has a robust corpus that is broad basally and tapers distally; the myxal area has a strong spiniform process and an extensive pad of small spinules. The claw is slender, with short basal barb and ventral spinules. Fine caudal rami are present (Fig. 5D) but do not extend beyond the distal trunk margin (visible through the cuticle).

##### Taxonomic remarks.

*Clavellomimus* includes four species: *C. macruri* (Hansen, 1923), *C. delamarei* (Nunes-Ruivo, 1953), *C. berecynthia* (Leigh-Sharpe, 1936), and *C. intermedius* (Quidor, 1906). Only *C. macruri* resembles the present specimens in terms of cephalothorax and trunk morphology, whereas the remaining species differ in trunk shape, cephalothorax–trunk proportions, and maxillary morphology.

The specimens from *M. holotrachys* have a relatively long cephalothorax and trunk; the latter is approximately 60% of the cephalothorax length, whereas it is 83–88% in *C. macruri*. The length of the maxilla reached 16% of the cephalothorax length. The armature of the endopod differs in the relative size of elements 2 and 3. Compared with *C. macruri*, the maxilliped shows a more developed pad of spinules in the myxal area. The trunks of the present specimens are cylindrical throughout, whereas those of *C. macruri* are flattened. A short genital process is present in *C. macruri* but is absent in our specimens. The fine caudal rami visible through the cuticle do not extend beyond the trunk margin, which is a distinctive feature of the specimens from *M. holotrachys*.

Although minor differences exist with *C. macruri*, these specimens are tentatively identified as *Clavellomimus macruri* until more material is available, particularly for DNA comparison. Further study is needed to clarify the relationships among *Clavellomimus* species, as their current composition remains uncertain.

### Phylogenetic data

**Figure 6.**
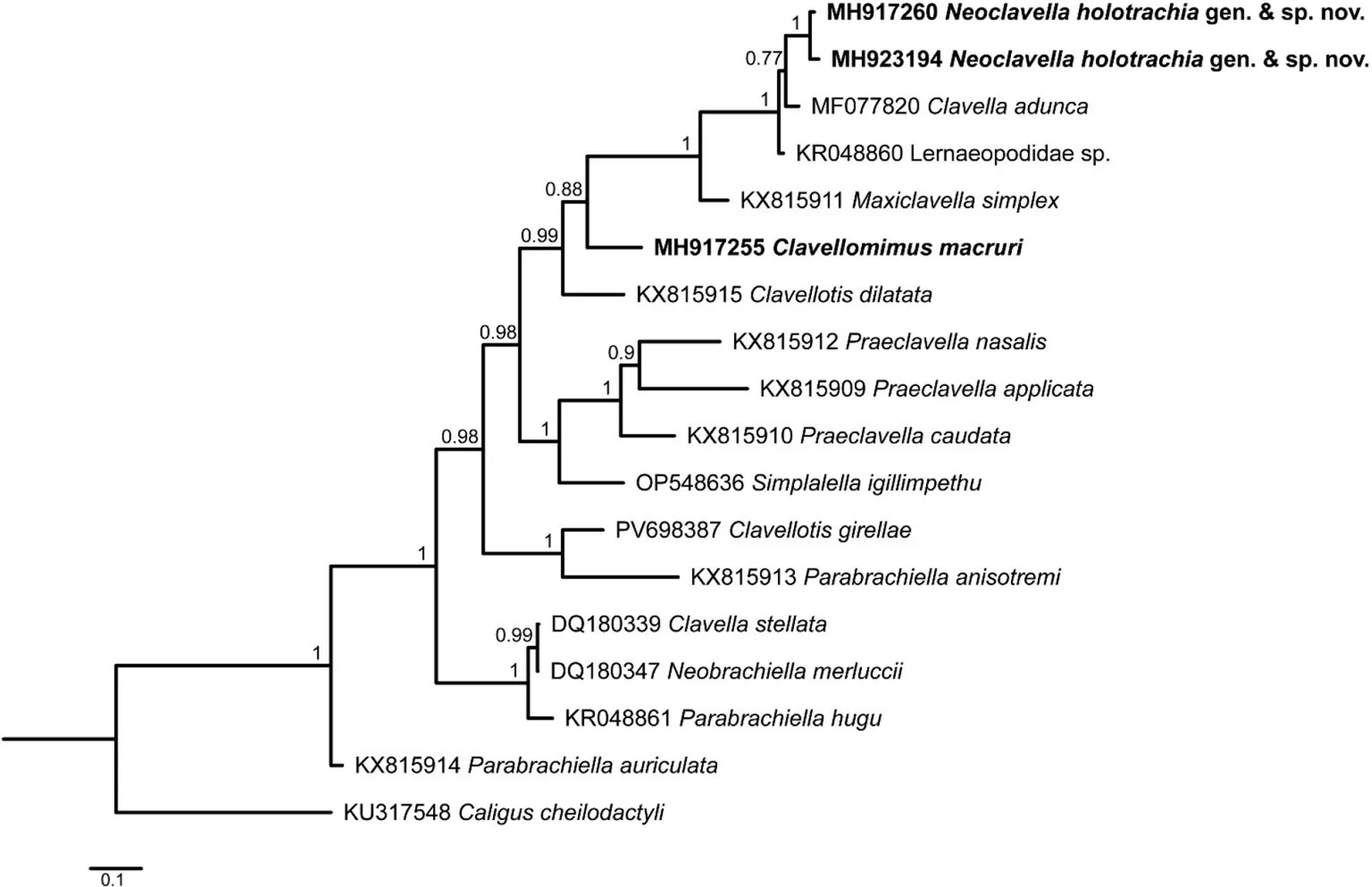
Phylogenetic tree inferred using Bayesian Inference derived from the 28s rDNA gene dataset. Numbers at nodes represent posterior probability (<90% are not shown). New sequences in bold.

The scarcity of material obtained from deep water and the poor fixation of some specimens usually makes it difficult to obtain good samples for molecular analysis. Therefore, few sequences were analyzed, but they were still useful as a complement to the morphological analysis and species delimitation. Two 28S rDNA sequences of 699 and 703 bp were obtained for *Neoclavella holotrachia* gen. nov. et sp. nov., and one of 677 bp was obtained for *Clavellomimus macruri*. These sequences were used to retrieve homologous sequences deposited in GenBank. The resulting phylogram is shown in Fig. 6. The new genus clusters with *Maxiclavella simplex*, Lernaeopodidae gen. sp., and *C. adunca* (PP = 1.00), with the latter being the sister genus of *Neoclavella* gen. nov. (PP = 0.77). *Clavellomimus macruri* clusters with *Clavellotis dilatata* in a weakly supported node (PP = 0.88).

The p distance values based on the 28S rDNA dataset (Table 4) show that *Neoclavella holotrachia* gen. nov. et sp. nov. has differences with *C. adunca* (4.1–4.7%, 15–24 bp) and Lernaeopodidae gen. sp. (6.2–6.8%, 28 bp). *Clavellomimus macruri* is different from *Clavellotis girellae* (15.3%, 81 bp) and *Clavellotis dilatata* (15.5%, 106 bp).

**Table 4.** Genetic divergence and nucleotide difference based on 28S rDNA among *Neoclavella holotrachia* gen. nov. et sp. nov. with other Lernaeopodidae species used in the phylogenetic analysis. Below the diagonal are the genetic distance estimated using uncorrected p-distances with gaps/missing data in MEGA X, and above the nucleotide differences. Newly generated sequences are shown in bold.

			1	2	3	4	5	6	7	8	9	10	11	12	13	14	15	16	17	18
1	**MH917260**	***Neoclavella holotrachia* gen. nov. et sp. nov**.		**15**	**23**	**44**	**107**	**110**	**134**	**160**	**167**	**173**	**175**	**178**	**178**	**186**	**195**	**206**	**218**	**242**
2	**MH923194**	***Neoclavella holotrachia* gen. nov. et sp. nov**.	**2.1**		**26**	**48**	**103**	**109**	**134**	**160**	**165**	**170**	**170**	**176**	**176**	**182**	**194**	**204**	**212**	**243**
3	MF077820	*Clavella adunca*	**4.1**	**4.7**		13	90	73	94	94	91	103	92	87	87	94	103	118	126	163
4	KR048860	Lernaeopodidae sp.	**6.2**	**6.8**	2.3		101	106	133	151	182	200	183	186	186	198	206	207	224	247
5	PV698387	*Clavellotis girellae*	**20.2**	**19.4**	17.5	19.0		97	77	81	76	66	80	84	84	77	93	107	67	163
6	KX815911	*Maxiclavella simplex*	**15.8**	**15.7**	13.2	14.6	18.2		118	132	177	165	191	181	181	203	201	204	222	252
7	OP548636	*Simplalella igillimpethu*	**22.4**	**22.3**	17.3	22.1	14.6	19.6		106	114	106	110	118	118	94	133	123	149	205
8	MH917255	*Clavellomimus macruri*	**24.1**	**24.2**	17.2	22.3	15.3	19.5	18.0		144	106	144	142	142	136	154	182	177	239
9	KX815914	*Parabrachiella auriculata*	**24.7**	**24.5**	16.8	25.9	14.8	25.2	19.8	22.0		167	170	153	153	180	162	182	173	180
10	KX815915	*Clavellotis dilatata*	**24.7**	**24.5**	18.6	27.5	12.2	22.8	17.4	15.5	23.7		182	173	173	178	186	177	211	264
11	KX815912	*Praeclavella nasalis*	**25.4**	**24.8**	16.9	25.5	15.0	26.7	18.2	21.5	24.6	25.2		179	179	137	192	130	197	250
12	DQ180339	*Clavella stellata*	**25.7**	**25.5**	15.8	25.8	15.8	25.1	19.8	20.9	21.9	23.9	25.2		1	199	44	202	188	243
13	DQ180347	*Parabrachiella merluccii*	**25.7**	**25.5**	15.8	25.8	15.8	25.1	19.8	20.9	21.9	23.9	25.2	0.1		199	43	202	187	243
14	KX815910	*Praeclavella caudata*	**26.7**	**26.3**	17.1	27.3	14.4	28.0	15.4	20.1	25.8	24.4	18.8	27.7	27.7		211	133	207	255
15	KR048861	*Parabrachiella hugu*	**27.9**	**27.9**	18.6	28.3	17.3	27.7	21.9	22.6	23.0	25.3	26.7	6.1	5.9	29.0		203	192	240
16	KX815909	*Praeclavella applicata*	**30.3**	**30.1**	21.5	30.0	19.9	29.5	20.1	27.0	27.4	25.4	18.6	29.4	29.4	18.9	29.3		218	265
17	KX815913	*Parabrachiella anisotremi*	**31.5**	**30.7**	23.0	31.1	12.4	30.9	24.7	26.2	24.8	28.9	27.4	26.1	26.0	28.5	26.4	31.3		262
18	KU317548	*Caligus cheilodactyli* *	**35.7**	**36.1**	29.6	35.9	30.6	36.6	34.4	35.5	26.7	38.2	36.9	35.4	35.4	37.2	34.8	38.9	38.2	

* Outgroup.

**Figure 7.**
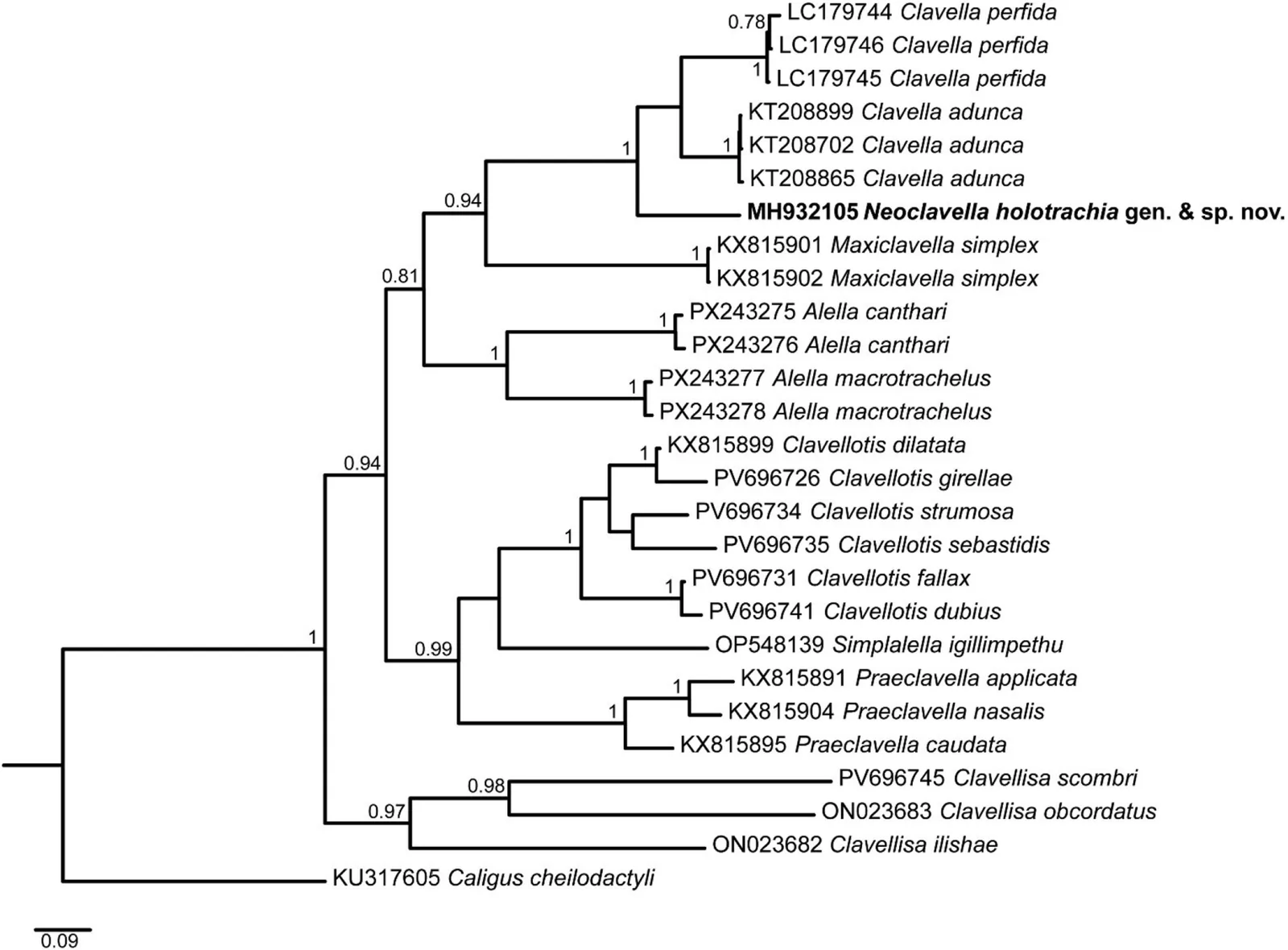
Phylogenetic tree inferred using Bayesian Inference derived from the cytochrome c oxidase subunit I (COI) gene dataset. Numbers at nodes represent posterior probability (<90% are not shown). New sequences in bold.

One COI mtDNA sequence (417 bp) was obtained for *Neoclavella holotrachia* gen. nov. et sp. nov., and homologous sequences were retrieved from GenBank. The resulting phylogram is shown in Fig. 7 and our specimen appears as the sister genus of a well-supported node (PP = 1.00) formed by *C. adunca* and *C. perfida*. The posterior probability of this clade is also high (PP = 1.00) with *Maxiclavella simplex*. The p distance values (Table 5) indicate that *Neoclavella holotrachia* gen.& sp. nov. is closely related to *C. adunca* (18.0–18.2%, 75–76 bp) and *C. perfida* (17.3–18.2%, 72–76 bp).

**Table 5.** Genetic divergence and nucleotide differences based on COI mtDNA among *Neoclavella holotrachia* gen. nov. et sp. nov. with other Lernaeopodidae species used in the phylogenetic analysis. Below the diagonal are the genetic distance estimated using uncorrected p-distances with gaps/missing data in MEGA X, and above the nucleotide differences. Newly generated sequences are shown in bold.

			**1**	**2**	**3**	**4**	**5**	**6**	**7**	**8**	**9**	**10**	**11**	**12**	**13**	**14**	**15**	**16**	**17**	**18**	**19**	**20**	**21**	**22**	**23**	**24**	**25**	**26**	**27**
1	MH932105	***Neoclavella holotrachia* gen. nov. et sp. nov**.		**72**	**73**	**76**	**75**	**75**	**76**	**86**	**88**	**87**	**88**	**88**	**88**	**90**	**97**	**97**	**98**	**100**	**100**	**104**	**104**	**105**	**111**	**112**	**113**	**122**	**111**
2	LC179746	*Clavella perfida*	**17.3**		5	13	103	102	104	142	147	139	138	161	161	134	152	134	126	150	144	158	155	118	167	151	154	129	168
3	LC179745	*Clavella perfida*	**17.5**	0.7		14	104	103	103	141	147	139	138	164	164	132	152	132	126	150	142	156	156	120	166	151	155	130	168
4	LC179744	*Clavella perfida*	**18.2**	1.9	2.0		107	106	106	141	148	139	138	161	161	130	148	129	123	146	146	159	156	122	167	152	155	133	168
5	KT208702	*Clavella adunca*	**18.0**	15.4	15.6	16.0		1	5	134	145	147	149	158	158	149	155	153	156	154	156	172	164	127	159	157	168	131	193
6	KT208899	*Clavella adunca*	**18.0**	15.3	15.4	15.9	0.1		4	133	144	146	148	157	157	148	154	152	155	153	155	173	163	127	158	156	167	131	193
7	KT208865	*Clavella adunca*	**18.2**	15.6	15.4	15.9	0.7	0.6		135	146	147	149	159	159	147	154	151	157	153	156	172	161	129	157	153	166	129	192
8	PX243276	*Alella canthari*	**20.8**	23.0	22.8	22.8	22.0	21.8	22.1		13	101	99	154	155	124	124	126	128	130	129	138	138	122	140	134	136	133	139
9	PX243275	*Alella canthari*	**21.1**	21.7	21.7	21.9	21.7	21.6	21.9	2.1		100	102	166	167	135	128	130	131	130	134	147	147	121	147	145	147	137	153
10	PX243277	*Alella macrotrachelus*	**20.9**	20.7	20.7	20.7	22.1	22.0	22.1	16.3	15.0		9	146	146	113	108	111	108	108	130	149	132	112	140	122	138	126	140
11	PX243278	*Alella macrotrachelus*	**21.1**	20.2	20.2	20.2	22.3	22.2	22.3	15.9	15.0	1.3		144	144	116	107	112	109	109	126	152	133	110	140	122	137	129	136
12	KX815902	*Maxiclavella simplex*	**21.1**	23.6	24.0	23.6	23.7	23.5	23.8	24.8	24.4	21.7	20.7		2	142	149	142	143	147	146	172	156	133	163	167	152	148	174
13	KX815901	*Maxiclavella simplex*	**21.1**	23.6	24.0	23.6	23.7	23.5	23.8	25.0	24.5	21.7	20.8	0.3		143	149	142	143	148	147	172	157	134	163	168	151	149	175
14	KX815904	*Praeclavella nasalis*	**21.6**	20.9	20.6	20.3	23.5	23.4	23.2	21.5	20.9	17.9	18.0	22.0	22.2		108	38	57	106	114	147	116	100	134	139	119	141	152
15	PV696731	*Clavellotis fallax*	**23.3**	22.3	22.3	21.7	23.2	23.1	23.1	20.0	18.8	16.1	15.4	21.4	21.5	16.7		108	108	17	81	153	118	79	94	134	95	130	146
16	KX815891	*Praeclavella applicate*	**23.3**	21.0	20.7	20.2	24.0	23.9	23.7	20.6	20.3	17.4	17.5	22.2	22.2	6.3	16.9		67	108	111	142	124	98	127	134	125	129	151
17	KX815895	*Praeclavella caudata*	**23.5**	19.4	19.4	18.9	24.3	24.1	24.4	20.6	20.3	16.6	16.7	21.9	21.9	9.4	16.5	10.5		112	112	148	121	101	135	140	122	135	160
18	PV696741	*Clavellotis dubius*	**24.0**	23.4	23.4	22.7	24.3	24.1	24.1	20.9	20.4	16.8	16.8	22.6	22.7	17.7	2.6	17.0	17.4		86	152	118	80	89	133	95	131	148
19	KX815899	*Clavellotis dilatata*	**24.0**	21.1	20.8	21.4	23.4	23.2	23.4	20.8	19.7	19.3	18.2	20.6	20.8	17.7	11.7	17.4	17.1	13.2		146	126	35	89	146	76	134	159
20	O-023683	*Clavellisa obcordatus*	**24.9**	25.6	25.2	25.7	27.8	28.0	27.8	24.6	23.8	24.2	24.6	27.8	27.8	23.8	24.8	24.1	24.9	26.0	23.6		148	135	160	144	148	138	168
21	OP548139	*Simplalella igillimpethu*	**24.9**	23.7	23.9	23.9	25.1	24.9	24.6	22.7	22.5	20.2	20.3	23.9	24.0	18.7	18.0	19.5	18.8	18.6	19.3	24.4		106	138	139	123	130	168
22	PV696726	*Clavellotis girellae*	**25.2**	22.0	22.3	22.7	23.6	23.6	24.0	23.0	22.5	20.9	20.5	24.8	25.0	18.6	14.7	18.2	18.8	14.9	6.5	25.3	19.7		86	137	67	128	133
23	PV696735	*Clavellotis sebastidis*	**26.6**	24.6	24.4	24.6	23.9	23.8	23.6	22.7	21.8	20.9	20.5	23.8	23.8	21.0	13.7	20.0	20.7	13.9	13.0	26.0	21.2	16.1		153	86	140	182
24	PV696745	*Clavellisa scombri*	**26.9**	24.2	24.2	24.3	25.4	25.3	24.8	23.9	23.1	19.9	19.3	26.3	26.7	22.1	21.2	22.8	23.6	22.8	23.2	23.3	23.0	25.7	24.6		153	131	155
25	PV696734	*Clavellotis strumosa*	**27.2**	22.6	22.8	22.8	25.3	25.1	25.0	22.0	21.6	20.6	19.7	21.5	21.8	18.5	13.7	19.6	18.7	14.6	11.0	24.0	18.9	12.5	12.6	24.2		143	167
26	O-023682	*Clavellisa ilishae*	**29.3**	23.3	23.5	24.0	23.6	23.6	23.3	26.6	24.7	22.7	23.3	26.7	26.9	25.5	23.5	24.5	25.3	25.0	24.2	24.9	23.9	25.3	25.3	23.6	25.9		136
27	KU317605	*Caligus cheilodactyli* *	**26.6**	24.9	24.9	24.9	29.3	29.3	29.2	22.7	22.8	21.1	19.8	25.3	25.6	23.9	21.3	24.0	24.8	23.2	23.3	27.5	26.0	25.2	26.8	24.7	24.3	24.5	

* Outgroup.

## Discussion

Species differentiation within *Clavella* has long been problematic, mainly because several descriptions were inaccurate or incomplete, particularly regarding appendages such as the antennule and antenna. Furthermore, the appendages exhibit distinct conformations that have often been misinterpreted; for example, Kabata (1963) observed errors in the description of *Clavella gracilis* Hansen, 1923, currently *Praeclavella* (*Praeclavella gracilis* [Castro-Romero et al. 2022]). Since the study by Kabata (1963), several *Clavella* species have been described (Castro and Baeza 1985; Ho 1993; Castro 1994; Castro and González 2009), some of which have uniramous antennae and belong to *Clavella*, while others with biramous antennae were later transferred to *Praeclavella* and *Maxiclavella* (Castro-Romero et al. 2022).

A clear example of misidentification is provided by Kabata and Gusev (1966), who studied specimens from two host species, *Trematomus loennbergi* and *Macrourus whitsoni*. These specimens differed in their secondary dentition: P1, S1, P1, S1, P1, S1, B2 in the specimens from *T. loennbergi*, vs P1, P1, P1, S1, P1, S1, B2 in those from *M. whitsoni*. Differences were also noted in trunk shape, which was suborbicular in the first host and oblong in the second. Such variation cannot be explained solely by intraspecific variability.

The specimens examined in this study, which are parasitic on *M. holotrachys*, differ strongly from *C. adunca* (previously reported from the same host and other macrourids) in terms of the antennular armature, antennal rami, mandible dentition, trunk shape, and the cephalothorax–trunk relationship. These clear morphological differences indicate that the present specimens from *M. holotrachys* do not correspond to *C. adunca*. Moreover, there may be more than one *Clavella*-branch species or genus parasitizing macrourid hosts.

The morphological differences between our specimens from *M. holotrachys* and *C. adunca* from *Gadus morhua* Linnaeus are corroborated by genetic divergence based on both COI mtDNA and 28S rDNA. This confirms that they are not conspecific and suggests that they belong to different genera. The 28S rDNA gene shows low divergence, as expected given its conservative nature. The relatively low evolutionary rate of 28S rDNA explains the reduced genetic divergence among the genera of Lernaeopodidae compared with the higher divergence observed in the COI for the caligid *Lepeophtheirus* spp. (González et al. 2016).

Similarly, the COI divergence levels between *Neoclavella holotrachia* gen. nov. et sp. nov. and other lernaeopodid species and genera are sufficient to justify the establishment of a new genus within the *Clavella* branch. Similar levels of genetic differentiation have been reported among genera of the *Clavella* and *Brachiella* branches (e.g., *C. adunca*, *Praeclavella applicata*, *P. caudata*, and *Parabrachiella* spp.; Castro-Romero et al. 2022).

*Clavella adunca* has a complex taxonomic history involving several subspecies and a long list of synonyms (Nuñes-Ruivo 1957; Kabata 1960, 1963, 1988). The results of the present study suggest that many records of *C. adunca* from different fish families (Gadidae, Macrouridae, etc.), habitats (deep vs shallow waters), and regions (North Atlantic, North Pacific, South Pacific, etc.) are misidentified. For instance, *C. adunca* reported on *Eleginops maclovinus* by Sepúlveda et al. (2004) and Henríquez et al. (2011) could correspond to *Praeclavella chiloensis* (formerly *Clavella chiloensis* Castro, 1994, found on *E. maclovinus*). However, without detailed morphological study, especially of the antennal structure, these distinctions are difficult to make. Reports of *C. adunca* on *Macrourus* species (Kabata and Gusev 1966; Münster et al. 2016) should therefore be reassessed.

Thus, previous records of *C. adunca* from *M. holotrachys* may be erroneous, or alternatively, more than one lernaeopodid species could parasitize this host. The coexistence of two lernaeopodid species on a single host is known; for example, *Praeclavella fortis* (Castro and González 2009) and *P. singularis* (Castro and González 2009) both parasitize *Nezumia pulchella* (Pequeño, 1971), and *Praeclavella longicauda* (Ho 1993) and *Clavella okamurai* Ho, 1993, are both found on *Coryphaenoides marginatus*. Additional material from other localities will allow for the clarification of the status of *Clavella*-branch species on macrourids, which are clearly distinct from *C. adunca* and other *Clavella* species.

## Conclusions

This study provides a description of a new genus of lernaeopodid copepods associated with deep-sea macrourid fishes, *Neoclavella* gen. nov. and a description of *Neoclavella holotrachia* gen. nov. et sp. nov. The combined morphological and molecular evidence (COI mtDNA and 28S rDNA) demonstrates that these specimens are not conspecific with *Clavella adunca*, supporting their placement in a distinct genus within the *Clavella* branch.

This study also highlights the problematic status of *C. adunca*, which likely represents a complex of cryptic or misidentified species across multiple host families, habitats, and geographic regions. Several previous records, particularly from macrourid hosts, should be critically re-evaluated using detailed morphological and molecular approaches.

## Supplementary Material

XML Treatment for Neoclavella

XML Treatment for Neoclavella holotrachia

XML Treatment for Clavellomimus macruri

## Additional information

### Artificial Intelligence (AI) use

The authors accept full responsibility for the content of the manuscript, including the disclosure of any use of AI.

No AI tools were used in the preparation of this manuscript.

### Author ORCIDs

R. Castro-Romero https://orcid.org/0000-0001-5358-4554

M.M. Montes https://orcid.org/0000-0002-7177-333X

L.A. Ñacari https://orcid.org/0000-0001-9692-8476

F. Sepulveda https://orcid.org/0000-0002-7876-7231

M.E. Oliva https://orcid.org/0000-0003-1759-2797

### Data availability

All of the data that support the findings of this study are available in the main text.
